# Factors Associated with Viral Load Suppression Among People Living with HIV on Antiretroviral Therapy in Bunia, Northeastern Democratic Republic of Congo

**DOI:** 10.3390/diseases14070238

**Published:** 2026-07-02

**Authors:** Alex Liripa Kwendra, Augustin Mouinga-Ondeme, Jéordy Dimitri Engone-Ondo, Roger Buju Tsedha, Justin Byaruhanga Ngona, Salomon Batina Agasa, Herman Chelo Ngadjole, Ivan S. Mfouo-Tynga, Zacharie Tsongo Kibendelwa

**Affiliations:** 1 Département de Médecine Interne, Infectiologie Tropicale, Faculté de Médecine, Université de Bunia, Bunia 292, Democratic Republic of the Congo; 2 Programme National de Lutte Contre le VIH/SIDA-IST-Hépatites Virales, Bunia 185, Democratic Republic of the Congo; 3 Unité des Infections Rétrovirales et Pathologies Associées, Centre International de Recherches Médicales de Franceville (CIRMF), Franceville P.O. Box 769, Gabon; 4 Département de Santé Publique, Faculté de Médecine, Université de Bunia, Bunia 292, Democratic Republic of the Congo; 5 Laboratoire de VIH/SIDA, Hôpital Général de Référence de Bunia, Bunia 238, Democratic Republic of the Congo; 6 Département de Médecine Interne, Universitaires de Kisangani, Kisangani 2012, Democratic Republic of the Congo; 7 Département de la Santé Publique, Université de Goma, Goma 2204, Democratic Republic of the Congo

**Keywords:** HIV, antiretroviral therapy (ART), viral load (VL), suppression, associated factors, Northeastern DRC

## Abstract

Background: The fight against HIV in the Democratic Republic of Congo (DRC) is hindered by systemic challenges; notably, limited access to HIV viral load (VL) testing. Achieving VL suppression is the primary global target for eliminating HIV as a public health threat by 2030. This study aimed to determine the rate of VL suppression and identify associated factors among people living with HIV (PLHIV) receiving antiretroviral therapy (ART) in Bunia. Methods: A descriptive and analytical cross-sectional study was conducted among PLHIV receiving care at treatment sites in Bunia over a period of 11 months. Participants were selected using a two-stage sampling approach, consisting of non-random quota sampling followed by simple random sampling. The primary endpoint was the proportion of PLHIV achieving VL suppression, while associated factors were identified using multiple logistic regression analysis. Results: Overall, 603 PLHIV were enrolled, including 180 males (29.9%) and 423 females (70.1%). The median age was 40 years (IQR: 32–48), median duration on ART was 38 months (IQR: 15–91), and VL suppression was achieved by 75% of the participants. While this indicates significant progress, it remains below the UNAIDS 95-95-95 targets. Adherence to antiretroviral therapy (aOR = 139.43; 95% CI: 66.63–291.76; *p* < 0.001) and being female (aOR = 2.15; 95% CI: 1.02–4.53; *p* = 0.044) emerged as independent predictors of virological success. Conclusions: Enhancing access to VL testing and optimizing ART adherence support strategies are critical to improving VL suppression rates in Bunia. Addressing these gaps is essential for the DRC to align with the global health objectives.

## 1. Introduction

The Human Immunodeficiency Virus (HIV), the causative agent of Acquired Immunodeficiency Syndrome (AIDS), remains a preeminent global public health challenge, particularly within low- and middle-income countries [1]. UNAIDS estimates indicate that, since the inception of the epidemic, approximately 91.4 million people have contracted HIV, with 44.1 million having succumbed to AIDS-related illnesses [2]. By the end of 2024, approximately 40.8 million people were living with HIV (PLHIV) globally. Notably, 65% of this population resides in the WHO African Region. While 31.6 million PLHIV now have access to life-saving antiretroviral therapy (ART), nearly a quarter of those infected remain without treatment [1,2]. To provide a coordinated response to the HIV epidemic, the World Health Organization (WHO), the Global Fund, and UNAIDS have developed global strategies aligned with Target 3.3 of the Sustainable Development Goals (SDGs), which aims to end the HIV/AIDS epidemic as a public health threat by 2030 [3]. These strategies are anchored by the 95-95-95 targets: ensuring that 95% of all people living with HIV (PLHIV) are diagnosed, 95% of those diagnosed receive sustained antiretroviral therapy (ART), and 95% of those on treatment achieve viral load (VL) suppression. Reaching these benchmarks is critical both to optimizing individual clinical outcomes and to eliminating HIV transmission within communities [4].

Beyond clinical adherence, PLHIV require consistent biological monitoring through viral load (VL) testing to verify the efficacy of antiretroviral therapy (ART); indeed, VL suppression serves as the most reliable indicator of treatment success [5,6]. In accordance with the 2021 WHO consolidated guidelines, virological suppression was defined as a viral load below 1000 copies/mL. This threshold serves as a critical indicator that antiretroviral therapy (ART) is effectively inhibiting viral replication and preventing clinical progression. Furthermore, the 1000 copies/mL benchmark is particularly well-suited for resource-limited settings, where the use of dried blood spot (DBS) testing and other field-based diagnostic tools is essential for maintaining high-quality HIV monitoring. Substantial evidence confirms that suppression is crucial for improving individual health, and preventing mother-to-child transmission [6]. Globally, the expansion of ART coverage from 25% in 2010 to 77% in 2024 has resulted in a significant decline in HIV-related morbidity and mortality [2]. Conversely, virological failure may emerge when patients lack consistent access to medication or fail to adhere to prescribed regimens. Systemic barriers, including pharmaceutical stockouts, treatment interruptions, suboptimal prescribing practices, and pervasive stigma and discrimination, remain primary drivers of virological failure within healthcare systems [5].

In the Democratic Republic of Congo (DRC), the HIV epidemic is categorized as generalized, with a national adult prevalence of 0.7% [7]. However, significant disparities exist across demographics: prevalence is estimated at 2.8% among pregnant women and 0.8% among the youth population (aged 15–24) [7,8]. Furthermore, women bear a disproportionate burden, with a seroprevalence rate of 1.2%, compared to 0.7% among men [8]. The Democratic Republic of Congo (DRC) has fully committed to the global vision of eliminating AIDS as a public health threat, primarily through the 95-95-95 targets integrated into its 2023–2027 National Strategic Plan [8,9]. As part of this strategy, the country has adopted dolutegravir (DTG)-based regimens as the preferred first-line treatment for HIV-positive patients [8]. National reports from 2024 indicate significant progress: 85% of PLHIV were aware of their status, 98% of those diagnosed had access to ART, and a viral suppression rate of 87% was achieved among those on treatment [10]. Bunia, the capital of Ituri Province, serves as a critical focal point for these efforts. HIV care services have been operational in the city since 2006, and Bunia currently manages an active ART caseload that accounts for approximately 40% of the entire provincial total [11].

However, the quality of care in this region is significantly compromised by regional instability and armed conflict, which have contributed to a high loss to follow-up (LTFU) rate of 11.37% among PLHIV [11,12]. Furthermore, clinical outcomes are hindered by severely limited access to viral load testing (2%) and a low viral suppression rate (72.8%), both of which drive higher rates of treatment failure and HIV-related mortality. These challenges were underscored by a prospective cohort study conducted in Bunia between 2019 and 2021 which reported a suppression rate of only 72.8% [12]. This figure is notably lower than the national average of 87% [11] and falls significantly short of the global average of 94% [2]. Prior data from this region, collected between 2019 and 2021, primarily focused on the transition from Efavirenz-based regimens, noted for their low genetic barrier to resistance, to the newly implemented Dolutegravir (DTG)-based therapy. While DTG is engineered to offer a significantly higher genetic barrier, there has been no formal evaluation of viral suppression rates or real-world outcomes has been conducted with the DTG-based regimen in Bunia in the five years since this programmatic shift. Thus, our study provides the first contemporary assessment of virological outcomes under the current standard of care within this specific humanitarian and resource-limited landscape.

Consequently, in resource-limited settings like the DRC, where genotypic resistance testing recommended by the WHO for monitoring primary and secondary ARV resistance is currently unavailable, viral load monitoring becomes even more indispensable [13]. This diagnostic tool remains the primary mechanism for assessing therapeutic efficacy, identifying virological failure, and guiding critical clinical decisions [8,13]. Achieving a suppressed or undetectable viral load is the definitive goal of any HIV control program; it optimizes individual health outcomes and curtails community-level transmission, thereby accelerating progress toward ending the epidemic [5]. To this end, the present study aimed to determine the current rate of viral load suppression in Bunia and identify its associated factors, providing the evidence base needed to develop more effective strategies for reaching global targets.

## 2. Materials and Methods

### 2.1. Study Setting and Population

**Figure 1:**
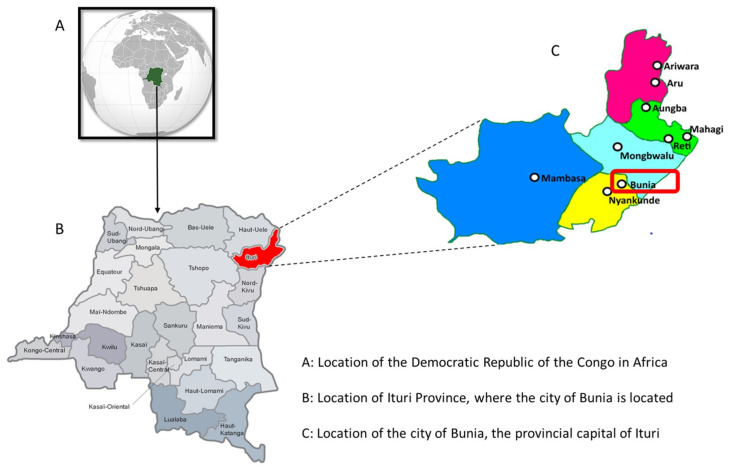
Inclusion site of Bunia, the capital city of the province of Ituri, Northeast of the Democratic Republic of Congo (DRC) in Africa.

Between December 2024 and October 2025, we conducted a descriptive and analytical cross-sectional study among people living with HIV (PLHIV) receiving antiretroviral therapy (ART) in Bunia, the capital of Ituri Province in the Democratic Republic of the Congo (DRC) (Figure 1). The study included PLHIV who had been on ART for at least six months and provided informed consent. Participants were recruited from 20 healthcare facilities (HCFs) providing comprehensive HIV care in accordance with national guidelines and the WHO public health approach for low-resource settings. The sample size was determined using the following formula for a finite population:

n = [N × Z^2^ × p × (1 − p)/[{N × E^2^ + Z^2^ × p × (1 − p)}]

where n: required sample size, N: total population of PLHIV on ART in the 20 HCFs, Z: 95% confidence level, p: expected proportion (50%), and E: the acceptable margin of error (5%).

While the initial sample size calculation required a minimum of 384 participants (assuming a 50% expected proportion at a 95% confidence level), we increased the final enrollment to 603 participants to enhance the statistical power of the study. This expanded cohort significantly improved the precision of our estimates, reducing the margin of error from 5% to approximately 4% (3.99%) while maintaining the 95% confidence interval. This larger sample size ensures a more robust analysis of the predictors associated with virological suppression in the study area.

We employed a two-stage sampling method. In the first stage, the sample size per stratum was determined using non-random quota sampling, based on 2023 DHIS2 data from the selected healthcare facilities (HCFs). The quota for each HCF was calculated by multiplying the total sample size by the proportion of each stratum. In the second stage, participants were selected from ART registries via simple random sampling according to the HCF quotas. A comprehensive list of eligible participants was compiled, from which 603 individuals were selected using the random number generator in OpenEpi v3.01. To minimize the impact of loss to follow-up, active tracing was conducted through home visits and telephone calls. Any selected participant who was absent or declined to participate was replaced by the next eligible individual on the randomized list to maintain the established quotas.

### 2.2. Inclusion and Exclusion Criteria

This study enrolled individuals living with HIV who had been receiving antiretroviral therapy (ART) for at least six months at the selected healthcare facilities. Written informed consent was obtained from all adult participants, while parental or legal guardian consent supplemented by minor assent was obtained for participants under 18 years of age. Exclusion criteria included individuals who had been transferred from other facilities, those with incomplete medical records, and any individuals who declined to participate or were unable to provide informed consent.

### 2.3. Data Collection

Data were collected using a standardized, pre-tested questionnaire administered through face-to-face interviews, supplemented by a retrospective review of medical records. The ‘pre-test’ mentioned was not an evaluation of a newly developed psychometric tool, but rather a feasibility pilot conducted with the first ten participants. This step was taken to verify that the questions were interpreted consistently and to ensure the overall reliability of the data collection process. This is a standard quality control measure to minimize information bias. The primary outcome was virological suppression, defined as a plasma viral load of less than 1000 copies/mL after at least six months of antiretroviral therapy (ART). According to the World Health Organization (WHO), this threshold serves as a robust indicator of therapeutic efficacy, confirming that the treatment is successfully inhibiting viral replication. Furthermore, this benchmark is specifically optimized for resource-limited settings, as it is compatible with field-based diagnostic tools such as dried blood spot (DBS) testing, which are vital for expanding monitoring in low-income countries [5,6]. The following categories of variables were collected:

Socio-demographic variables: Gender, age, educational attainment, employment status, marital status, and place of residence.

Clinical and biological variables: WHO clinical stage, specific ARV regimen, duration of ART, HIV viral load, treatment adherence, and the presence of current opportunistic infections.

Therapeutic adherence is defined as the extent to which a patient’s behavior including antiretroviral intake and lifestyle modifications aligns with medical recommendations. In this study, adherence was assessed using a combination of participants self-reports and pill counts. Thus, participants who reported missing no ARV doses within the past 30 days and no prescription renewal appointments during the three months preceding the survey were classified as adherent [14].

We monitored opportunistic infections corresponding to WHO clinical stages 2, 3, and 4. These infections were included if they were either ongoing or had occurred within the three months preceding the survey, provided they were diagnosed by trained physicians or nurse practitioners [15]. All diagnoses were reviewed and validated during the therapeutic committee meetings at each respective healthcare facility (HCF).

### 2.4. Laboratory Methods

For each participant, 6 mL of whole blood was collected in an EDTA tube and transported within 3 h to the HIV laboratory at the Bunia General Reference Hospital. Upon arrival, blood samples were processed via centrifugation (Thermo Scientific Medifuge, Osterode am Harz, Germany), 2000 rpm for 15 min. Plasma samples were analyzed for viral load (VL) within 24 h of collection. The quantification of VL was performed using the Xpert^®^ HIV-1 Viral Load test (Cepheid^®^, Sunnyvale, CA, USA) on the GeneXpert^®^ platform, strictly following the manufacturer’s protocols. The assay has a lower limit of detection of 40 copies/mL.

### 2.5. Statistical Analysis

Data were analyzed using R software version 4.6.0. Descriptive statistics were computed to summarize the sociodemographic and clinical characteristics of the participants, with categorical variables presented as frequencies and percentages.

Bivariate analyses were performed using binomial logistic regression to evaluate factors associated with viral suppression, yielding crude odds ratios (ORs), 95% confidence intervals (95% CIs), and *p*-values. To enhance the robustness and interpretability of the regression models, categories with limited sample sizes were merged; specifically, postgraduate education was combined with university education, and polygamous marital status was grouped with the married category.

Variables with *p*-values less than 0.20 in the bivariate analysis, alongside clinically relevant variables, were entered into the multivariable logistic regression model. Adjusted odds ratios (aORs), 95% CIs, and *p*-values were reported to identify independent predictors of viral suppression. The final multivariable model included WHO clinical stage, adherence to antiretroviral therapy, history of opportunistic infections, and duration of antiretroviral therapy. Multicollinearity was assessed using the Variance Inflation Factor (VIF) and tolerance values. VIF values ranged from 1.01 to 1.15, while tolerance values ranged from 0.868 to 0.992, indicating no evidence of problematic multicollinearity among the independent variables included in the model. Statistical significance was defined as a two-sided *p*-value less than 0.05.

## 3. Results

### 3.1. Study Population Characteristics

Between December 2024 and October 2025, a total of 603 people living with HIV (PLHIV) who had been receiving antiretroviral therapy (ART) for at least six months were enrolled in the study. All participants were recruited from 20 pre-selected healthcare facilities (HCFs). The study population was predominantly female, accounting for 70.1% (n = 423) of the total, while males represented 29.9% (n = 180) of the participants (Table 1).

### 3.2. Socio-Demographic Characteristics

The study population spanned a wide age range, with a median age of 40 years (IQR: 32–48). Participants aged 48 years and older constituted the largest age group, representing 30.0% of the sample. Regarding employment, over half of the participants (50.7%, n = 306) were employed, while 49.3% (n = 297) were unemployed. The majority of the cohort reported having at least a basic education (78.3%), specifically at the primary (45.8%) or secondary (32.5%) levels. Additionally, 31.5% of the participants were single. The vast majority of the study population resided in urban areas (90.0%), with only 10.0% living in rural settings (Table 1).

### 3.3. Clinical and Biological Characteristics

At the initiation of antiretroviral therapy (ART), a significant proportion of participants (36.5%) were at an advanced WHO clinical stage. Following initiation, the vast majority (95.0%) were prescribed a regimen of TDF + 3TC + DTG. The median duration of ART for the entire cohort was 38 months (IQR: 15–91). Over half of the participants (62.2%) had been receiving their current ART regimen for at least 24 months prior to study enrollment A large proportion of participants did not have opportunistic infection at the time of the survey. The treatment adherence rate was 72.5% in this study. Laboratory analysis revealed a viral suppression rate (<1000 copies/mL) of 75.0% (n = 452), while 25.0% (n = 151) of participants presented with a viral load ≥1000 copies/mL (Table 2).

### 3.4. Factors Associated with Viral Suppression (Bivariate Analysis)

In the bivariate analysis, female participants were significantly more likely to achieve viral suppression than male participants (OR = 1.69; 95%; CI: 1.14–2.49; *p* = 0.008). Regarding age, participants aged ≥48 years were significantly more likely to achieve viral suppression compared with those aged 28–37 years (OR = 1.94; 95% CI: 1.17–3.23; *p* = 0.011) (Table 3). Moreover, three clinical factors were significantly associated with a higher probability of viral suppression. Treatment adherence was the main predictor of success; patients adhering to treatment were 127.54 times more likely to achieve optimal viral suppression than those not adhering (OR = 127.54; 95% CI: 66.63–244.12; *p* < 0.001). Furthermore, patients without opportunistic infections had 3.70 times higher odds of achieving viral suppression compared to those with opportunistic infections (OR = 3.70; 95% CI: 2.50–5.26; *p* < 0.001). Early initiation of antiretroviral therapy was also a significant predictive factor: patients initiated at WHO stage I (OR = 3.47; 95% CI: 2.12–5.67; *p* < 0.001) and at stage II (OR = 2.08; 95% CI: 1.33–3.26; *p* = 0.001) had significantly higher chances of suppression than those initiated at later stages (Table 4).

### 3.5. Multivariate Logistic Regression

In the multivariable logistic regression model, adherence to antiretroviral therapy (aOR = 139.43; 95% CI: 66.63–291.76; *p* < 0.001) and female gender (aOR = 2.15; 95% CI: 1.02–4.53; *p* = 0.044) remained independently associated with viral suppression after adjusting for WHO clinical stage, opportunistic infections, duration of ART, and age group (Table 5).

## 4. Discussion

The World Health Organization (WHO) emphasizes that achieving HIV viral suppression is essential for improving individual clinical outcomes and preventing vertical transmission of HIV. As a cornerstone of the global strategy to end the AIDS epidemic, viral suppression transforms HIV from a terminal illness into a manageable chronic condition while simultaneously safeguarding public health [5,6]. In the present study, we evaluated the viral load (VL) suppression rate and analyzed the factors associated with treatment success among patients receiving antiretroviral therapy (ART) in Bunia, DRC.

Our study revealed a viral load (VL) suppression rate of 75.0%. This figure is lower than those reported in recent studies conducted in the Congolese provinces of Kinshasa and Haut-Katanga, where rates ranged from 81.0% to 84.9% [16,17,18]. It is also lower than the national average of 87.0% reported in 2024 [10] and remains significantly below the UNAIDS target of 95% [6], and ≥95% reported in a systematic review and meta-analysis of 13 low and middle-income countries [19]. Regionally, our results are lower than the 78.7% reported in Brazzaville [20] and the 85.7% observed in Uganda [21] but higher than the 59% found in Kenya [22]. Although our suppression rate is comparable to the 74.0% reported in a South African study [23], it is higher than the 57.9% observed in Bukavu (South Kivu) [24] and the 72.8% reported in Bunia [12]. These discrepancies highlight the considerable challenges faced by HIV programs in resource-limited settings when striving to achieve global targets. In particular, compared to the findings of Buju et al. (2019–2021) [12], who observed a significant increase in virological suppression in the Bunia region, this modest but positive trend is likely attributable to strengthened support for treatment adherence at the community level and the implementation of advanced HIV care protocols [8,25] at Bunia General Referral Hospital over the past five years.

After adjusting for confounding variables, two factors remain significantly and independently associated with viral load suppression (VL). Our findings identify ART adherence as the primary predictor of virological success. Adherent participants were 135.25 times more likely to achieve viral suppression compared to those who did not. This strong association is consistent with the global evidence synthesized in recent WHO reports on drug resistance and strategic directions, as well as multiple regional studies [5,13,21,23,26,27,28,29]. The WHO emphasizes that intensive adherence counseling during both ART initiation and follow-up is essential to achieving and maintaining an undetectable viral load, the ultimate objective of antiretroviral therapy [5]. However, our study observed a concerningly low treatment adherence rate of 72.5%. This shortfall underscores the significant barriers to achieving the third UNAIDS 95% target in our specific context. Our findings are supported by Mandro (2022), who reported a 28.0% prevalence of non-adherence in the same region [30]. In the Bunia area, this poor performance can be largely attributed to the persistence of armed conflict, which triggers recurrent waves of population displacement.

Our findings in Bunia align with global observations regarding the high genetic barrier of Dolutegravir. For instance, a study conducted in Brazil [31] has similarly reported a rapid virological suppression following the transition to DTG-based ART. By comparing the ‘Advanced HIV Care’ model in Bunia with global standards, it becomes evident that community-based intensification is an universally effective strategy for overcoming structural barriers to adherence, regardless of the geographic region.

In the Bunia region, these suboptimal outcomes are further exacerbated by ongoing armed conflict, which triggers recurring waves of population displacement, road closures, and the looting or destruction of healthcare facilities. Such instability leads to critical disruptions in the supply chain for antiretroviral (ARV) drugs, viral load testing kits, and other essential consumables. These systemic failures frequently result in forced treatment interruptions and the suspension of routine viral load monitoring. Patients rendered non-adherent due to these exogenous drug shortages are at heightened risk of viral rebound and the subsequent emergence of antiretroviral-resistant strains.

In this study, being female was significantly associated with viral load (VL) suppression. This finding contributes to an ongoing discussion in the literature, where the association between gender and virological outcomes remains inconsistent across different regions. While several studies conducted in similar sub-Saharan contexts reported no significant differences in suppression rates between men and women [17,21,26], our results align with data indicating more favorable outcomes for female patients [18]. This gender-based disparity in Bunia may be attributed to greater health-seeking behavior among women or more frequent interaction with the healthcare system through maternal and child health services. Conversely, men in these settings often face distinct barriers to adherence, including labor-related migration, heightened social stigma, and lower rates of timely diagnosis.

Several limitations must be considered when interpreting the findings of this study. First, we did not evaluate structural barriers to healthcare accessibility, such as geographical distance to facilities or the direct and indirect costs of care, both of which may significantly influence consistent patient engagement. Second, the study did not formally quantify the impact of HIV-related stigma or the specific effects of conflict-related population displacement on longitudinal follow-up. Finally, the absence of detailed data regarding patients’ prior history of antiretroviral therapy (ART) exposure including potential previous treatment interruptions constitutes a limitation, as these factors are known to influence current virological outcomes. Future research incorporating these socio-environmental and clinical variables would provide a more comprehensive understanding of the drivers of viral suppression in such a complex humanitarian landscape.

## 5. Conclusions

In conclusion, while the 2024 UNAIDS report demonstrates significant progress globally in controlling the epidemic, our findings show that resource-limited and conflict-affected settings, such as Bunia, face specific challenges in achieving the final 95-95-95 targets. Adherence to antiretroviral treatment and being female have been shown to be independent predictors of virological success.

This finding underscores that behavioral consistency remains the primary driver of treatment success. However, in a context of limited routine viral surveillance and a lack of drug resistance testing, the “test and treat” model must be reinforced by robust community support systems. Therefore, we recommend the systematic assessment of treatment adherence and the provision of intensive support, when necessary, at every routine follow-up visit. Furthermore, there is a critical need for tailored, differentiated programmatic interventions designed to dismantle the logistical, psychosocial, and cultural barriers driving these sex- and gender-based disparities. Similarly, a reliable supply chain and strengthened health infrastructure are essential to ensure it can withstand external shocks related to regional instability. Strengthening these structural pillars is crucial to guaranteeing that progress toward achieving global HIV goals leaves no one behind.

## Figures and Tables

**Table 1:** Socio-demographic characteristics of participants.

Characteristics	N = 603	%
Gender		
Female	423	70.1
Male	180	29.9
Age group (years)		
<18	46	7.6
18–27	54	9.0
28–37	171	28.4
38–47	151	25.0
≥48	181	30.0
Employment status		
Employed	306	50.7
Unemployed	297	49.3
Education level		
Illiterate	85	14.1
Postgraduate	3	0.5
Primary	276	45.8
Secondary	196	32.5
University	43	7.1
Marital status		
Single	190	31.5
Divorced/Separated	43	7.1
Married	126	20.9
Polygamous marriage	3	0.5
Cohabiting	124	20.6
Widowed	117	19.4
Residence		
Rural	60	10.0
Urban	543	90.0

N: total population.

**Table 2:** Clinical and biological characteristics of participants.

Characteristics	N = 603	%
WHO clinical stage		
Stage I	191	31.7
Stage II	181	30.0
Stage III	220	36.5
Stage IV	11	1.8
ART regimen		
ABC-3TC-DTG	1	0.2
ABC-3TC-DTG 50 mg	2	0.3
ABC-3TC-DTG (pediatric)	22	3.6
ABC-3TC-LPV/r	1	0.2
AZT-3TC-DTG	3	0.5
TDF-3TC-DTG	573	95.0
TDF-3TC-EFV	1	0.2
Duration of ART (months)		
6–11	86	14.3
12–24	142	23.5
>24	375	62.2
Opportunistic infection		
No	429	71.1
Yes	174	28.9
Viral load (copies/mL)		
>1000	151	25.0
<1000	452	75.0
ART adherence		
No	166	27.5
Yes	437	72.5

N: total population; ART: Antiretroviral treatment; ABC: Abacavir; DTG: Dolutegravir; TDF: Tenofovir; 3TC: Lamivudine; AZT: Zidovudine; EFV: Efavirenz.

**Table 3:** Association between socio-demographic factors and HIV viral load suppression.

Suppression of Viral Load						
Variables	No (%)	Yes (%)	Total (%)	OR	[95% CI]	*p*-Value
Gender						
Male	58 (9.6)	122 (20.2)	180 (29.9)	Ref	-	-
Female	93(15.4)	330 (54.7)	423 (70.1)	1.69	[1.14, 2.49]	0.008
Age group (years)						
28–37	49 (8.1)	122 (20.2)	171 (28.4)	Ref	-	-
<18	17 (2.8)	29 (4.8)	46 (7.6)	0.69	[0.35, 1.36]	0.279
18–27	18 (3.0)	36 (6.0)	54 (9.0)	0.80	[0.42, 1.55]	0.513
38–47	36 (6.0)	115 (19.1)	151 (25.0)	1.28	[0.78, 2.12]	0.329
≥48	31 (5.1)	150 (24.9)	181 (30.0)	1.94	[1.17, 3.23]	0.011
Residence						
Rural	16 (10.6)	44 (9.7)	60 (10.0)	Ref	-	-
Urban	135 (89.4)	408 (90.3)	543 (90.0)	1.10	[0.60, 2.01]	0.760
Level of education						
Illiterate	19 (12.6)	66 (14.6)	85 (14.1)	Ref	-	-
Primary	68 (45.0)	208 (46.0)	276 (45.8)	0.88	[0.49, 1.57]	0.667
Secondary	54 (35.8)	142 (31.4)	196 (32.5)	0.76	[0.42, 1.38]	0.362
University	10 (6.6)	36 (8.0)	46 (7.6)	1.04	[0.44, 2.47]	0.936
Marital status						
Single	50 (33.1)	140 (31.0)	190 (31.5)	Ref	-	-
Divorced/Separated	7 (4.6)	36 (8.0)	43 (7.1)	1.84	[0.77, 4.39]	0.172
Married	34 (22.5)	95 (21.1)	129 (21.4)	1.00	[0.60, 1.66]	0.994
Cohabiting	40 (26.5)	84 (18.6)	124 (20.6)	0.75	[0.46, 1.23]	0.256
Widowed	20 (13.2)	97 (21.5)	117 (19.4)	1.73	[0.97, 3.09]	0.063

OR: Odds Ratio; CI: Confidence Interval; Married and polygamous participants were grouped due to the small number of polygamous participants (n = 3); Due to the very small number of participants in the postgraduate category (n = 3), this category was merged with the university education group for statistical analysis.

**Table 4:** Association between clinical factors and HIV viral load suppression.

Suppression of Viral Load						
Variables	No (%)	Yes (%)	Total (%)	OR	[95% CI]	*p*-Value
WHO clinical phase						
III	80 (53.0)	140 (31.0)	220 (36.5)	Ref	-	-
I	27 (17.9)	164 (36.3)	191 (31,7)	3.47	[2.12–5.67]	<0.001
II	39 (25.8)	142 (31.4)	181 (30.0)	2.08	[1.33–3.26]	0.001
IV	5 (3.3)	6 (1.3)	11 (1.8)	0.69	[0.20–2.32]	0.544
Opportunistic infection						
No	75 (49.7)	354 (78.3)	429 (71.7)	3.70	[2.50–5.26]	<0.001
Yes	76 (50.3)	98 (21.7)	174 (28.9)	Ref	-	-
Duration of ART						
6 to 11	30 (19.9)	56 (12.4)	86 (14.3)	Ref	-	-
12 to 24	41 (27.2)	101 (22.3)	142 (23.5)	1.35	[0.76–2.40]	0.303
>24	80 (53.0)	295 (65.3)	375 (62.2)	1.98	[1.19–3.28]	0.009
Adherence to ART						
No	136 (90.1)	30 (6.6)	166 (27.5)	Ref	-	-
Yes	15 (9.9)	422 (93.4)	437 (72.5)	127.54	[66.63–244.12]	<0.001

OR: Odds Ratio; CI: Confidence Interval, ART: Antiretroviral Therapy.

**Table 5:** Multivariate logistic regression analysis of factors associated with VL suppression among PLHIV.

Variables	Category	aOR (95% CI)	*p*-Value
WHO stage	III	Ref	-
	I	2.06 (0.80–5.32)	0.137
	II	0.88 (0.35–1.94)	0.664
	IV	0.53 (0.04–7.56)	0.652
Adherence to ART	No	Ref	-
	Yes	139.43 (66.63–291.76)	<0.001
Opportunistic infections	Yes	Ref	
	No	0.92 (0.42–2.01)	0.831
Duration under ART (months)	6 to 11	Ref	-
	12 to 24	1.69 (0.57–4.97)	0.340
	>24	1.89 (0.71–5.02)	0.199
Gender	Male	Ref	-
	Female	2.15 (1.02–4.53)	0.044
Age group (years)	28–37	Ref	-
	<18	0.51 (0.13–1.94)	0.323
	18–27	0.52 (0.15–1.81)	0.307
	38–47	1.47 (0.61–3.54)	0.390
	≥48	1.77 (0.70–4.43)	0.225

aOR: adjusted Odds Ratio; CI: Confidence Interval; ART: Antiretroviral Therapy.

## Data Availability

All data supporting the reported results of this study are included in the manuscript. Additional information could be available upon request to the corresponding author.

## Abbreviations

The following abbreviations are used in this manuscript:

3TC	Lamivudine
ABC	Abacavir
AIDS	Acquired immunodeficiency syndrome
aOR	adjusted Odds Ratio
ART	Antiretroviral therapy
ARV	Antiretroviral
AZT	Zidovudine
CI	Confidence Interval
DRC	Democratic Republic of Congo
DTG	Dolutegravir
EDTA	Ethylene-diamine-tetra-acetic acid
EFV	Efavirenz
HCFs	Healthcare facilities
HIV	Human Immunodeficiency Virus
IQR	Interquartile Range
LTFU	Loss to follow-up
MTCT	Mother-to-child transmission
OIs	opportunistic infections
OR	Odds Ratio
PLHIV	People living with HIV
SDGs	Sustainable development goals
TDF	Tenofovir
UNAIDS	Join United Nations Programme on HIV/AIDS
VL	Viral Load
WHO	World Health Organization

## References

[1] WHO (2024). HIV.

[2] UNAIDS (2025). FACT SHEET 2025—Global HIV Statistics. https://www.unaids.org/sites/default/files/2025-07/UNAIDS_FactSheet_fr.pdf.

[3] WHO (2025). HIV-AIDS.

[4] WHO Global health sector strategies on, respectively, HIV, viral hepatitis and sexually transmitted infections for the period 2022–2030. https://apps.who.int/iris/?locale-attribute=fr&.

[5] WHO (2023). The Role of HIV Viral Suppression in Improving Individual Health and Reducing Transmission: Policy Brief.

[6] UNAIDS (2024). Untransmittable—Public Health and HIV Viral Load Suppression.

[7] ONUSIDA (2025). Fiche d’Information—Dernières Statistiques sur l’état de l’Épidémie de Sida.

[8] PNLS RDC (2025). Guide de Prise en Charge Intégrée du VIH en République Démocratique du Congo. https://www.pnmls.cd/documentation/uploads/guide-de-prise-en-charge-integree-du-vih-en-republique-democratique-du-congo.pdf.

[9] (2024). National HIV/AIDS Response Monitoring Report DRC. Kinshasa. https://www.pnmls.cd/documentation/uploads/RAPPORT%20GAM%202024.pdf.

[10] PNLS DRC (2025). 2024 Annual Report of Activities to Combat HIV&AIDS and STIs.

[11] PNLS Ituri (2025). 2024 Annual Report of Activities to Combat HIV&AIDS and STIs.

[12] Buju R.T., Akilimali P.Z., Kamangu E.N., Mesia G.K., Kayembe J.M.N., Situakibanza H.N. (2022). Predictors of viral non-suppression among patients living with HIV under dolutegravir in Bunia, Democratic Republic of Congo: A prospective cohort study. Int. J. Environ. Res. Public Health.

[13] WHO (2024). HIV Drug Resistance: Brief Report 2024.

[14] Cissouma A., Kassogué D., Dembélé G., Traoré M., Haidara D., Poma H., Kelema P., Diallo F., Dicko-Traoré F., Sacko K. (2021). L’Observance du Traitement Antirétroviral chez les Enfants Infectés par le VIH à l’Hôpital de Sikasso. Health Sci. Dis..

[15] Ekwaru J.P., Campbell J., Malamba S., Moore D.M., Were W., Mermin J. (2013). The effect of opportunistic illness on HIV RNA viral load and CD4+ T cell count among HIV-positive adults taking antiretroviral therapy. J. Int. AIDS Soc..

[16] Ewetola R., Shah G., Etheredge G., Maluantesa L., Waterfield K., Olivas M., Engetele E., Bijou M.B. (2023). Viral load suppression among patients receiving antiretroviral therapy in outpatient clinics in Democratic Republic of Congo. HIV AIDS Rev. Int. J. HIV-Relat. Probl..

[17] Mayasi Ngongo N., Kamangu Ntambwe E., Situakibanza Nani-Tuma H., Mbula Mambimbi M., Mandina Ndona M., Longokolo Mashi M., Izizag B.B., Lukiana T., Ossam J.O., Sonzi D.M. (2023). Human immunodeficiency virus viral load monitoring and rate of virologic suppression among patients receiving antiretroviral therapy in Democratic Republic of the Congo, 2013–2020. Open Forum Infectious Diseases.

[18] Shah G.H., Maluantesa L., Etheredge G.D., Waterfield K.C., Ikhile O., Beni R., Engetele E., Mulenga A. (2021). HIV viral suppression among people living with HIV on antiretroviral therapy in Haut-Katanga and Kinshasa provinces of Democratic Republic of Congo. Healthcare.

[19] Girón-Callejas A., Lorenzana R., Pickles M., Inzaule S., Jordan M.R., Diaz S., Vrinten C. (2025). High HIV viral suppression among adults receiving WHO-recommended first-line dolutegravir-based antiretroviral therapy in low-and middle-income countries: A systematic review and meta-analysis of programmatic evidence. AIDS Res. Ther..

[20] Ontsira Ngoyi E.N., Moyen N., Mieret T., Aloumba A., Ossibi Ibara B.R. (2025). Assessment of Viral Load Suppression in HIV-Infected Children in Brazzaville (Congo): Évaluation de la Suppression de la Charge Virale chez les Enfants Infectés par le VIH à Brazzaville (Congo). Health Sci. Dis..

[21] Wakooko P., Gavamukulya Y., Wandabwa J.N. (2020). Viral load suppression and associated factors among HIV patients on antiretroviral treatment in Bulambuli district, eastern Uganda: A retrospective cohort study. Infect. Dis. Res. Treat..

[22] Maina E.K., Mureithi H., Adan A.A., Muriuki J., Lwembe R.M., Bukusi E.A. (2020). Incidences and factors associated with viral suppression or rebound among HIV patients on combination antiretroviral therapy from three counties in Kenya. Int. J. Infect. Dis..

[23] Okonji E.F., Van Wyk B., Mukumbang F.C., Hughes G.D. (2021). Determinants of viral suppression among adolescents on antiretroviral treatment in Ehlanzeni district, South Africa: A cross-sectional analysis. AIDS Res. Ther..

[24] David W.S., Masudi B.N., Lubunga E.A., imanya Cubaka F., Zalufa M.A., Masemo D.B., Parvine B.B. (2023). Profil de la charge virale chez les per-sonnes vivant avec le virus de l’immunodéficience humaine suivies au centre de traitement ambulatoire de l’Hôpital Général de Référence de Panzi: Etude transversale descriptive. Kivu Med. J..

[25] World Health Organization (2020). Package of Care for Children and Adolescents with Advanced HIV Disease: Stop AIDS.

[26] Bagnan Tossa L., Houdégnonto I., Tohodjédé Y., Assogba Zohmaléto M. Factors Associated with Viral Load Suppression in Children and Adolescents Living with HIV/AIDS on Antiretroviral Treatment in the ZOU Department in 2023. https://www.sobeped.com/wp-content/uploads/2025/01/4BagnanCV_VIH-enfants_adolescents-Zou25-31.pdf.

[27] Opoku S., Sakyi S.A., Ayisi-Boateng N.K., Enimil A.K., Senu E., Ansah R.O., Aning B.D., Ojuang D.A., Wekesa D.N., Ahmed F.O. (2022). Factors associated with viral suppression and rebound among adult HIV patients on treatment: A retrospective study in Ghana. AIDS Res. Ther..

[28] Yumba Numbi G., Matanda Kapend S., Basema Muheha M., Mwadi Kamalo B., Ndua Tshijik, Musung mbaz J., Wembulua B., Muyumba kiyana E., Ngoy nkulu D., Mwamba Mulumba C. (2025). Les déterminants associés à la suppression de la charge virale chez les personnes vivant avec le VIH sous traitement à base de Dolutégravir. Rev. Mali. Infect. Microbiol..

[29] UNAIDS Global AIDS Strategy 2021–2026, Ending Inequality, Ending AIDS, Geneva. https://www.unaids.org/sites/default/files/media_asset/global-AIDS-strategy-2021-2026_fr.pdf.

[30] Mandro C.N., Tibamwenda Y.B., Mosomo T.K., Wolyec S.T., Kibendelwa Z.T., Wembonyama S.O. (2023). Determinants of non-adherence to antiretroviral treatment among people living with HIV in the cities of Bunia and Goma: An analytical cross-sectional study. JMPHPR.

[31] Meireles M.V., Pascom A.R.P., Duarte E.C., McFarland W. (2019). Comparative effectiveness of first-line antiretroviral therapy: Results from a large real-world cohort after the implementation of dolutegravir. AIDS.

